# Successful management of extremely high‐output refractory congenital chylothorax with chemical pleurodesis using 4% povidone–iodine and propranolol: a case report

**DOI:** 10.1002/ccr3.1449

**Published:** 2018-02-27

**Authors:** Kathleen Borcyk, Ammar Kamil, Kristine Hagerty, Melissa Deer, Paul Tomich, Ann L. Anderson Berry

**Affiliations:** ^1^ Pediatrics University of Nebraska Medical Center Omaha Nebraska; ^2^ Pharmacy Nebraska Medicine Omaha Nebraska; ^3^ Obstetrics and Gynecology University of Nebraska Medical Center Omaha Nebraska

**Keywords:** Chylothorax, iodine, lymphangiectasia, pleurodesis, propranolol

## Abstract

First‐line therapy for congenital chylothorax is conservative treatment. However, surgical intervention or chemical pleurodesis is required for refractory cases. With all the concerns regarding its complications, povidone–iodin provided a successful management for a high‐output congenital chylothorax. However, renal and thyroid function must be monitored during treatment.

## Background

Congenital Chylothorax (CC) is the most common cause of pleural effusions in neonates. Treatment consists of medical management and drainage with expectation of spontaneous resolution. Cases with persistent or high‐volume output suffer from significant losses of proteins, electrolytes, fats, and lymphocytes. Surgical treatments, including chemical pleurodesis, have been used for refractory cases. A wide variety of chemicals have been used, each with its own complications. Pleurodesis with povidone–iodine was first reported in 2002 and has a 58–75% success rate in lower output cases. This treatment is controversial due to concerns for renal failure, hyperthyroidism, allergic reaction, and cardiorespiratory failure.

## Introduction

Chylothorax can be characterized by a lymphocyte‐rich pleural effusion with >110 mg/dL of triglycerides, ratios of pleural fluid to serum triglycerides and/or pleural fluid to serum cholesterol of greater than 1.0, or chylomicrons on lipoprotein analysis 1. This condition is rare with incidence reported as 1:5775 to 1:24,000, and a male to female ratio of 2:1 2, 3. Chylothoraces can be congenital, a result of a surgical complication or birth trauma, or idiopathic. Congenital chylothorax has a mortality rate of 20%, most commonly as a result of sepsis 3. Congenital chylothorax is often associated with chromosomal abnormalities, congenital heart disease, intrauterine infections, or lymphatic malformations; however, it can also be an isolated manifestation 2. First‐line treatment focuses on supportive care and conservative interventions. The success rate with conservative treatment alone has been reported to be as high as 80% 4. However, patients with persistent or high‐volume output are often refractory to first‐line therapy. These patients are also at increased risk for complications secondary to the significant losses of proteins, electrolytes, fats, and lymphocytes. For refractory cases, surgical intervention or chemical pleurodesis is required. In pediatric cases of refractory chylothorax, nonsurgical therapies are attractive. However, a lack of consensus on the preferred methods and protocols makes treatment for critically ill individuals difficult. This paper aims to present a case of isolated congenital chylothorax with extraordinarily high‐volume chylous leak that was resistant to conservative therapies but responded well to treatment with chemical pleurodesis using povidone–iodine and oral propranolol therapy.

## Case Report

**Figure 1:**
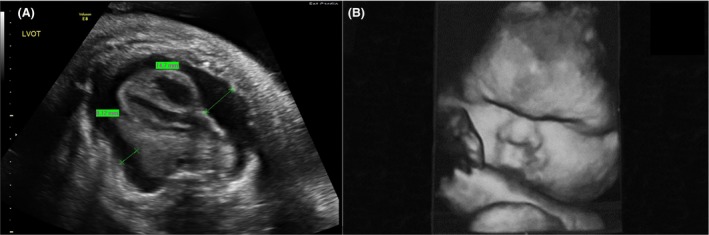
Ultrasound images illustrating a pericardial effusion (A) and overall edema (B).

**Figure 2:**
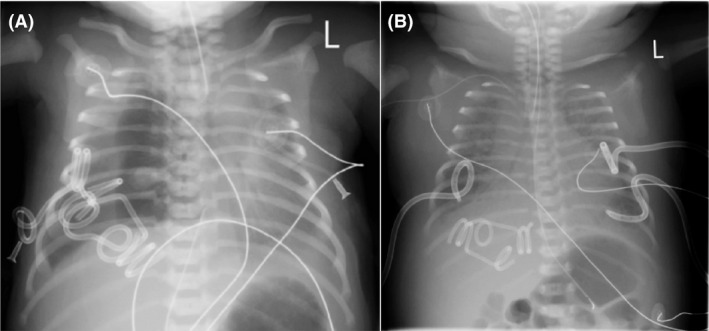
Chest radiograph at birth (A) and prior to pleurodesis (B).

**Figure 3:**
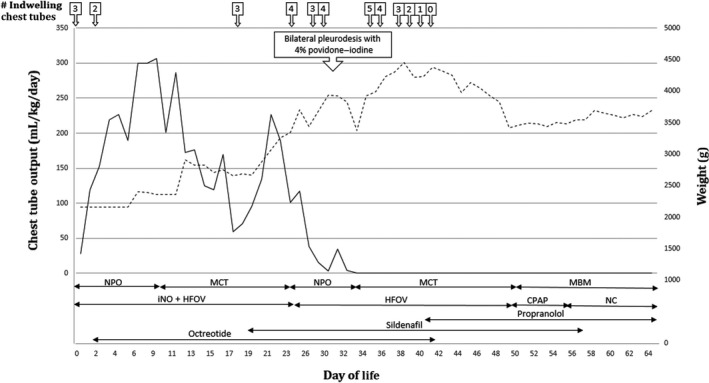
Pleural drainage and weight change after iodine instillation and propranolol therapy. Solid line is pleural drainage from chest tubes and dotted line is the patent's weight. NPO, nothing by mouth; MCT, medium‐chain triglyceride; MBM, maternal breastmilk; iNO, inhaled nitric oxide; HFOV, high‐flow oscillating ventilation; CPAP, continuous positive airway pressure; NC, nasal cannula.

A fetus of a healthy 36‐year‐old G5P3313 mother was first diagnosed with hydrops fetalis, pleural effusions, abdominal ascites, skin edema, and polyhydramnios at a referral community hospital at 31 weeks gestational age by ultrasound (see Fig. 1). The pregnancy had been uncomplicated to date. Three fetal thoracoamniotic shunts were subsequently placed in addition to an amnioreduction of 2000 mL at another referral center. Maternal screen for congenital infections and fetal–maternal hemorrhage was negative, and amniocentesis for karyotype and fluorescence in situ hybridization (FISH) was unremarkable (Table 1). Due to deteriorating condition, the 2165‐gram female neonate was born by cesarean section at 33‐week gestation at our institution, 2 weeks after the intrauterine thoracic shunts were placed. At delivery, APGARs were 4, 6, and 7, and the infant was intubated as part of resuscitation and stabilization. Due to severe respiratory distress, the infant was placed initially on conventional ventilation followed by high‐frequency oscillation (HFOV) with 20 parts per million of inhaled nitric oxide. Bilateral chest drainage catheters were placed less than one hour after delivery, and 28 mL of clear yellow fluid was drained from the left hemithorax. Initial pleural fluid analysis did not appear chylous, (see Table 2) but as the neonate was nourished, the fluid analysis became typical of a chylothorax. Initial serum measurements showed hypoproteinemia (1.6 g/dL) and hypoalbuminemia (<1.0). The neonate had substantial chest tube output, reaching as much as 306.5 mL/kg/day. To account for these losses, albumin, electrolytes, platelets, FFP, and pRBCs were given as needed on several occasions. Dopamine and dobutamine were also given for hypotension and oliguria that was due to renal hypoperfusion. Due to concerns for infection, the neonate was started on empiric antibiotics three separate times during her stay; these were discontinued once all cultures were negative from blood and pleural fluid. Renal, liver, and thyroid functions were monitored throughout her stay and remained normal. Octreotide, parenteral nutrition, and a medium‐chain triglyceride (MCT)‐enriched diet were trialed for control of chylothorax without improvement. Sildenafil was begun on DOL (day of life) 18. Despite some decrease in chest tube output while on sildenafil, the neonate became increasingly edematous and developed worsening respiratory status (Figure 2). On DOL 30 in light of progressive cardiorespiratory deterioration the decision was made to attempt bilateral pleurodesis with 6 mL of 4% povidone–iodine indwelling for 5 h. She tolerated the procedure well with fentanyl for analgesia. Follow‐up thyroid and renal studies were all normal. The chest tube output ceased after pleurodesis (Figure 3). By DOL 41, the chest tubes were all removed and there was no reaccumulation of fluid. A chest CAT SCAN on DOL 22 was inconclusive for lymphangiectasia. Due to continued edema and continued suspicion of lymphangiectasia not visualized on CT, propranolol was started on DOL 40 at 0.5 mg/kg/day Q8H and increased the dose to 2 mg/kg/day Q8H over 9 days. The patient diuresed, and her weight remained stable after beginning propranolol. She did not experience any episodes of hypoglycemia or bradycardia secondary to the propranolol. On DOL 50, the neonate was successfully extubated and was placed on CPAP. She was discharged home on DOL 64 with 125 mL oxygen via nasal cannula and propranolol with the intent to allow the infant to outgrow of the dose slowly. On follow‐up by age 6 months, the patient was no longer requiring oxygen and is growing and developing normally for her corrected age of 12 months. She slowly weaned off propranolol by 1 year and has not demonstrated any signs of recurrence of the pleural effusions. Her weight is in the 50th percentile, length between the 50th and 75th percentile, and head circumference in the 75th percentile on the IHDP low birth weight premature girls' growth curve. At a 6‐month evaluation, she is showing mild truncal hypotonia, but since that time this has resolved appears to be developing normally.

**Table 1:** Chromosomal and infectious work‐up

Amniocentesis	
Karyotype	Normal
FISH	Normal
Microarray	Normal
Toxoplasmosis PCR	Negative
Parvo B19 PCR	Negative
CMX PCR	Negative
Maternal Serum	
TORCH titers	Negative
RPR	Negative
Parvo B19 Antibody	Negative
Neonate Serum	
Blood and Surface HSV	Negative
Blood PCR Adenovirus	Negative
Blood PCR CMV	Negative
Urine CMV Culture	Negative
Lysosomal leukocyte panel,	Normal
Mucolipidoses II and II	Normal
Urine organic acid	Normal
Pleural fluid	
Pleural fluid viral and bacterial culture	Negative

**Table 2:** Pleural and serum fluid analysis

	DOL 0	DOL 2	DOL 4	DOL 8
Pleural fluid				
Albumin	–	<1.0	–	–
Cholesterol	–	8	–	–
Sodium	–	136	142	–
Potassium	–	2.4	2.5	–
Chloride	–	100	109	–
Glucose	–	67	–	82
Protein	–	<1.0	<1.0	2.2
Triglycerides	–	–	26	39
WBC	397	30	215	464
Lymphocyte %	70%	52%	90%	92%
Polys	2%	1%	2%	1%
Monos	28%	45%	7%	6%
Eos	0%	0%	1%	1%
RBC	<3000	<3000	<3000	<3000
Chylomicrons	–	–	–	Absent
Serum				
Sodium	137	117	136	136
Potassium	3.8	2.7	2.9	3.2
Chloride	100	88	108	107
Total protein	–	1.6	–	3.6
Albumin	–	<1.0	–	3

## Discussion

Congenital chylothorax is the most common form of pleural effusion in neonate and carries with it significant morbidity and mortality 5. This patient had several characteristics that are known to be associated with a poorer prognosis such as diagnosis prior to 33‐weeks gestation, mediastinal shift, hydrops fetalis, and persistent or high‐volume chylous effusions 6. Infants suffering from such conditions are at increased risk for pulmonary hypoplasia due to compression from the fluid, leading to respiratory distress or heart failure 1, 7. They are also at risk for malnutrition, infection, and death secondary to massive loss of proteins, electrolytes, fats, and lymphocytes. For this reason, it is imperative to identify and treat these patients early with attention to fluid, nutrition, and pharmacological management.

First‐line therapy focuses on supporting the patient and treating with conservative methods. As many as 75–80% of patients will have spontaneous resolution with supportive treatment alone 8. Improved outcomes are seen with the use of pressure‐controlled ventilation with positive end‐expiratory pressure (PEEP) or HFOV (utilized in this case) with or without inhaled nitric oxide 6. Either thoracentesis or placement of chest tubes can be used to alleviate the burden from the effusion and allow the lungs to expand, as was also seen with this patient. With the loss of nutrient‐rich and lymphocyte‐rich fluid, infants are at high risk for malnourishment and infection. It is important to replace such losses with the necessary albumin and electrolytes and to monitor the patient closely for any signs of infections. Lymphatic flow may be decreased with a low fat, high medium‐chain triglyceride (MCT) enteral diet; however, any enteral feeds have the potential to make the chylous fluid more viscous, impeding drainage of the effusions. In such cases, total parenteral nutrition may be a more beneficial alternative; neither of these methods was successful in this patient 9. Conservative interventions are also recommended as first‐line therapy for patients. Sildenafil and somatostatin or somatostatin analogs are the two primary means of conservative treatment. The proposed mechanism of action for the phosphodiesterase‐5 inhibitor, sildenafil, is an increase in nitric oxide which stimulates lymphangiogenesis and increases the clearance of lymph fluid 10. Somatostatin and somatostatin analogs such as octreotide, on the other hand, act on receptors in splanchnic vessels to decrease intestinal blood flow, gastrointestinal motility, and intestinal fat absorption, thereby decreasing lymphatic flow and accumulation of chyle in the thorax 11. The major complications related to somatostatin treatment are necrotizing enterocolitis from decreased splanchnic blood flow, increased liver enzymes, hyperglycemia, and transient hypothyroidism 2, 12.

While first‐line therapy alone is successful therapy for the majority of patients, in severe cases such as our patient, the condition becomes much more difficult to manage and treat. There currently is a lack of consensus on when more intensive therapies ought to be perused. The proposed indications focus on persistence of chyle production despite maximal treatment, large volumes of chyle production, associated complications, and overall clinical deterioration. Intensive treatments of persistent cases of chylothorax include surgical interventions and chemical pleurodesis. Surgical interventions include procedures such as thoracic duct ligation, fibrin glue, pleuroperitoneal shunts, mechanical surgical abrasion, and pleurectomy 1, 3, 9, 13. Due to our patient's extremely critical condition, it was determined that she was not a candidate for such procedures. When our patient's condition continued to deteriorate, the decision was made to pursue pleurodesis. The purpose of chemical pleurodesis is to infuse a substance into the pleural space that will cause an inflammatory reaction subsequent sclerosis, thereby closing off the source of chyle leakage. Several chemicals have been used for this procedure, including talc, *viscum album* extract, picibanil, minocycline, bleomycin, and povidone–iodine 14, 15, 16. Our patient had 4% iodine instilled bilaterally (sequentially) into her pleural cavity for 5 h. In other cases, 4–10% iodine has been used from one to five hours 17, 18. A lack of consensus on protocol for such a procedure has resulted in a great amount of variability in outcome. An analysis of 12 cases by Resch, et al. found a success rate of 58% and a survival rate of 75% 18. Serious complications that have occurred after pleurodesis with iodine include acute and chronic renal failure, respiratory distress, and hemodynamic and cardiorespiratory failure 18. Other potential side effects include hyperthyroidism via the Jod‐Basedow phenomenon, hypothyroidism, an allergic reaction, and pain 17, 19. It is believed that the risk of systemic toxicity and subsequent renal failure is highest in patients with pulmonary lymphangiectases 11, 20. It is hypothesized that this abnormality allows for greater systemic absorption that results in intoxication. It is for these reasons, that we suggest evaluation of infants for lymphangiectasis (by CT, definitive diagnosis can be made by lung biopsy) as a possible cause of the persistent chylothorax. Morbidity and mortality are higher in those infants found to have such abnormalities, and treatment with iodine should be cautioned. When iodine pleurodesis is used, it is important to monitor renal and thyroid function prior to and following pleurodesis so the appropriate interventions can be made.

The use of propranolol, the well‐known antihypertensive medication, has been serendipitously noted to control the growth of hemangiomas by Léauté‐Labrèze and her coworkers in 2008 in a large‐scale placebo‐controlled randomized clinical study while being in use for treatment of (congenital) heart disease in French children 21. As a consequence, oral propranolol has become the first‐line therapy for the treatment of hemangiomas 22 for its efficacy and safety 23.

The expression of beta2‐adrenergic respecters (*β*2‐ARs) had been identified in cases of infantile hemangioma that responded to propranolol treatment 24. As the activation of these receptors results in the synthesis of proangiogenic factors, such as vascular endothelial growth factor (VEGF) and basic fibroblast growth factor (bFGF), which activate proangiogenic cascades [extracellular signal‐related kinases/mitogen‐activated protein kinases (MAPK) cascade] promoting angiogenesis 25. Also, activation of *β*2‐ARs inhibits apoptosis mediated by Src tyrosine kinase, MAPK, and caspase cascades 24.

The statistically significant efficacy of propranolol and its recommended dosage (3 mg/kg/day) have been reported in a large‐scale placebo‐controlled randomized clinical study 8 As a consequence, oral propranolol has become the first‐line therapy for the treatment of IH 9.

While the literature on propranolol in lymphangiomas, still inconclusive; many reports have shown efficacy 26, 27, 28, 29, 30, 31, which had been related to the expression of *β*2‐ARs in the malformed lymphatic vessels 24. Propranolol showed improvement of symptoms in a subset of patients with lymphatic anomalies and limited the growth of congenital LMs in utero. Propranolol effects seemed to be dose‐dependent, with the lowest effective dose at 0.7 to 1 mg/kg/day. When propranolol was decreased or discontinued, qualitative experience and quantitative measures worsened 26.

We were unable to confirm a diagnosis in our patient of lymphangiomas by CT and given her clinical improvement after pleurodesis and propranolol; lung biopsy was not indicated. However, her response to propranolol suggested the presence of mixed vascular malformations 32. The decision to begin a trial of propranolol was influenced by our patient's significant generalized edema that worsened even after pleurodesis. It was not until the addition of propranolol that we witnessed a significant decrease in weight and improvement in her respiratory status. The choice to use 2 mg per kilogram was based on an article by Poralla et al. that demonstrated regression of lymphangiomatous lesions and improvement in the respiratory status of a preterm infant with generalized lymphangiectasia 30. In the treatment of hemangiomas, treatment with 3 mg per kilogram (kg) for 6 months was found to have a greater efficacy than 1 mg per kg 31. While our patient did have improvement with an intermediate dose of 2 mg/kg, we cannot say that it is superior to treatment with a higher dose.

We present a unique case of congenital chylothorax with high‐output volumes much larger than cases previously described and suspected lymphangiectasis that was successfully treated with intense supportive therapy, chemical pleurodesis with 4% povidone–iodine, and propranolol. Twelve months later, she is doing remarkably well despite her tenuous early hospital course. We comment on the morbidity of the disease and emphasize the importance of diligent monitoring of the patient's condition and prompt supportive therapy in order to have the best possible outcome, even in the most severe of cases.

## Conclusion

This case illustrates a high‐volume output of congenital chylothorax that was successfully managed with 4% povidone–iodine pleurodesis in a patient who was not a candidate for other surgical interventions. Meticulous fluid and nutritional management was critical for survival given the high‐volume losses. Renal and thyroid function must be followed during treatment with povidone–iodine. Adjunct therapy of propranolol was likely critical for success.

## Conflict of Interest

None declared.

## Authorship

KB: collect and interpreted the clinical data and perform drafting. AK: contributed in drafting, editing, and revision. KH: contributed in drafting. MD, and PT: contributed to patient care. ALAB: contributed to patient care, analysis of the data and final review of the manuscript.
